# Supplementary figures and images for: Comprehending the lack of access to maternal and neonatal emergency care: Designing solutions based on a space-time approach
Source: PLoS One. 2020 Jul 23;15(7):e0235954. doi: 10.1371/journal.pone.0235954 (PMC7377445; doi:10.1371/journal.pone.0235954)

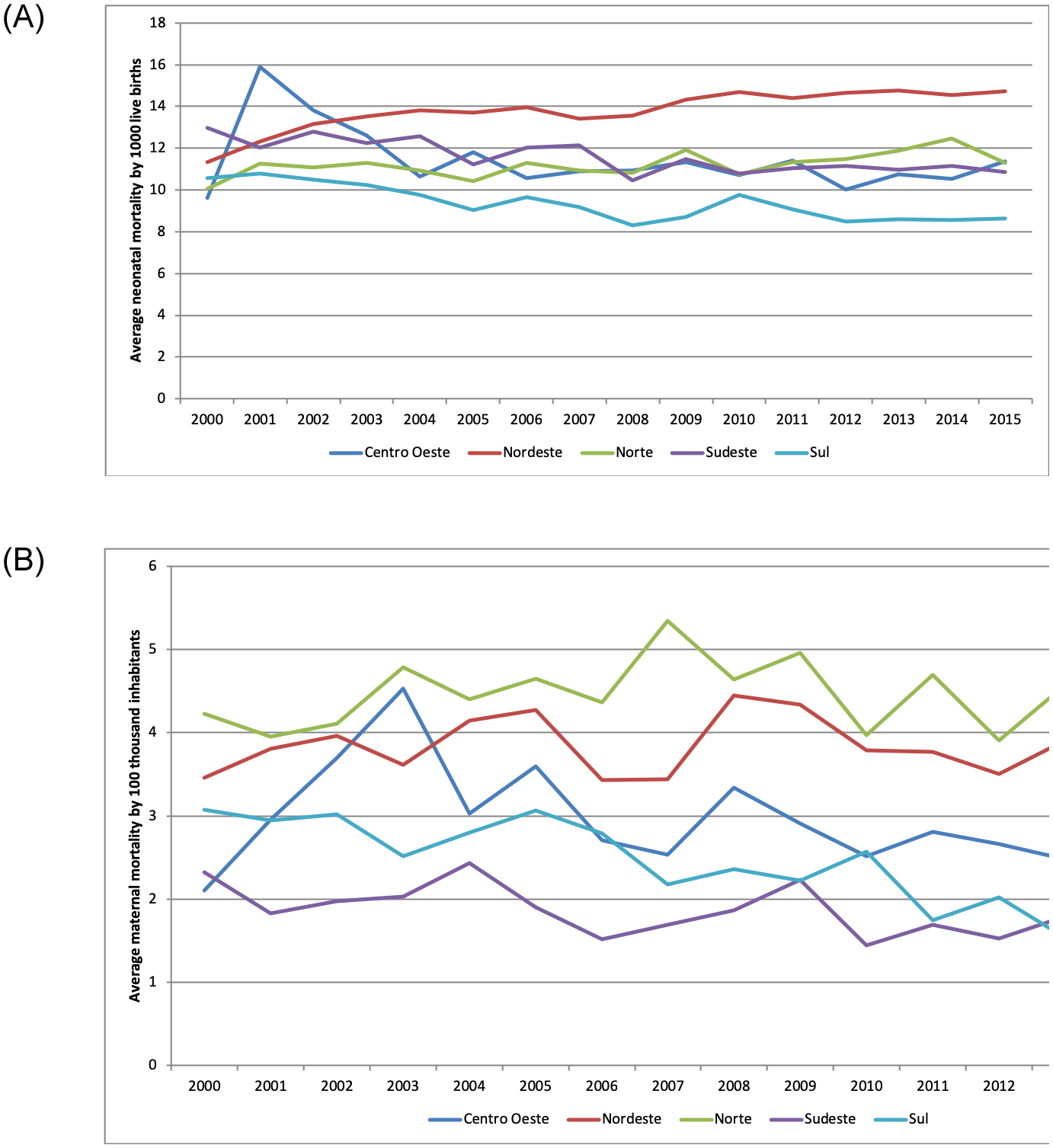

Supplement: S1 Fig — (TIF) [file pone.0235954.s001.tif]

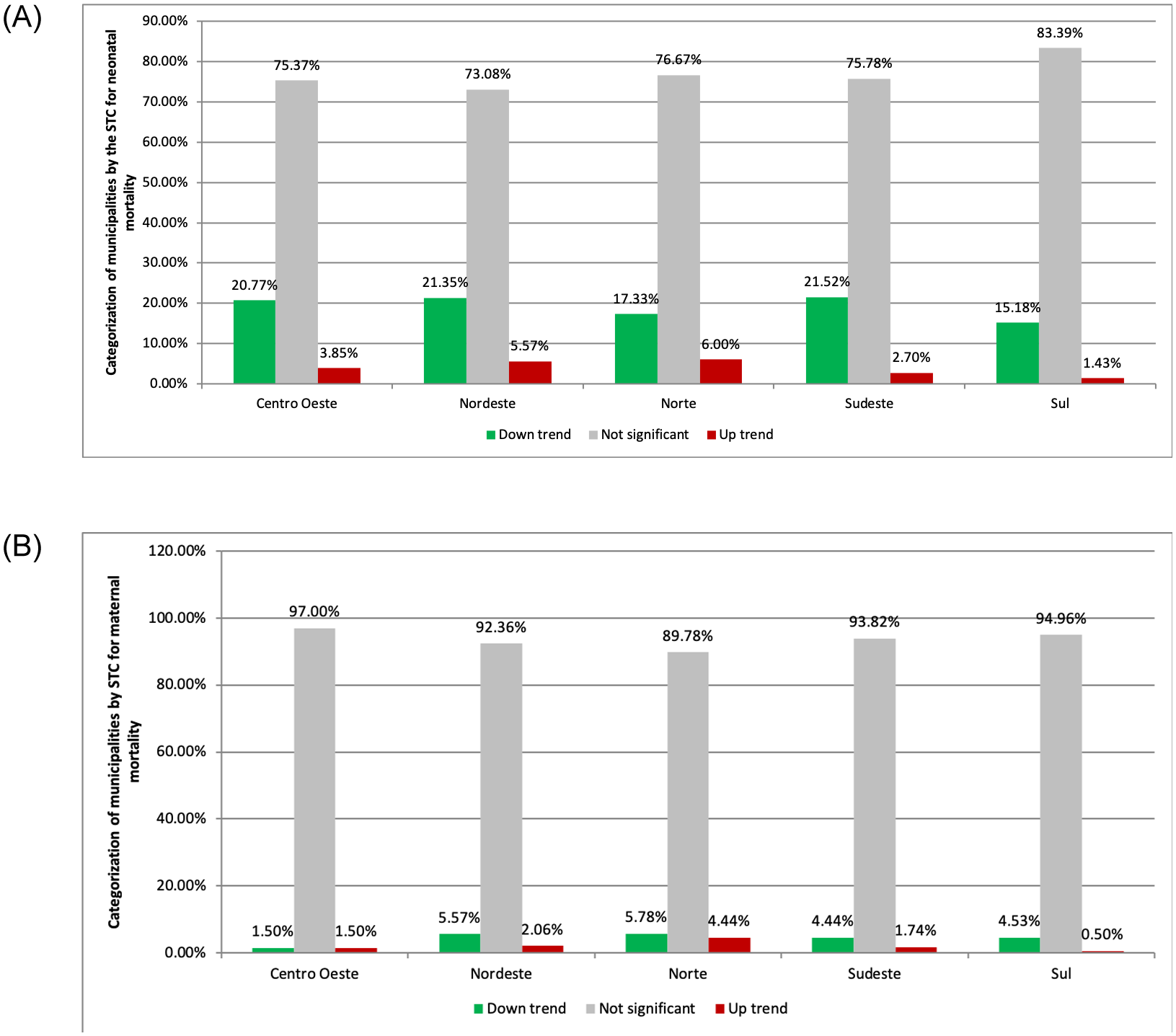

Supplement: S2 Fig — (TIF) [file pone.0235954.s002.tif]
